# Incidence Rate of Somatic Dysfunction in Previously Undiagnosed Spotted Fever Rickettsiosis: A Case Report

**DOI:** 10.7759/cureus.24416

**Published:** 2022-04-23

**Authors:** Mark D Unger, Joy L Palmer, Nichole M Thorsvik

**Affiliations:** 1 Osteopathic Neuromusculoskeletal Medicine, Liberty Mountain Medical Group, Lynchburg, USA

**Keywords:** vasculopathy, osteopathic structural examination, somatic dysfunction, osteopathic manipulative treatment, spotted fever rickettsiosis

## Abstract

Spotted Fever Rickettsiosis (SFR) is a systemic vasculopathy due to tick-borne rickettsial infection. Presenting symptoms and signs may be nonspecific or include the triad of fever, headache, and a rash. Established long-term complications of SFR include debilitating neuromusculoskeletal sequelae; however, no reports describe the incidence of somatic dysfunction (SD) in SFR. We present the first description of SD in previously undiagnosed SFR. Incidence of SD before diagnosis and after antibiotic therapy was assessed every seven weeks throughout 28 weeks of Osteopathic Neuromusculoskeletal Medicine (ONMM) care, including osteopathic manipulative treatment (OMT) administered twice a month on average. The patient presented with the chief complaint of worsening neck and back pain interfering with sleep. Other symptoms included blurry vision, right-hand weakness, a truncal rash, and absence of fevers. A 14-week trial of OMT failed to significantly decrease the incidence of SD compared to baseline. Extensive workup for an underlying condition revealed moderate axonal sensorimotor polyneuropathy and elevated rickettsial IgG titers. Doxycycline therapy was initiated alongside ongoing ONMM care. Incidence of SD over the 14-week post-antibiotic OMT period was significantly less than that assessed at baseline and during the OMT-only period. This case highlights the utility of periodic graphical assessment for monitoring SD response to OMT.

## Introduction

Spotted Fever Rickettsiosis (SFR) is a systemic vasculitis due to infection by one of several rickettsial pathogens [1,2]. Rocky Mountain Spotted Fever (RMSF) is the most severe rickettsial illness in the United States: mortality approaches 25% in untreated cases [1,3,4]. Approximately 50% of SFR cases occur in Arkansas, Missouri, North Carolina, Tennessee, and Virginia [2]. SFR is strongly suspected after a tick bite and presents with symptoms of fever, headache, and rash [5]. SFR begins after tick bite and dermal inoculation of rickettsial microorganisms [4]. These obligate intracellular bacteria spread via lymphatic channels and small-to-medium sized blood vessels and enter the intracellular compartment of endothelial cells or perivascular myocytes [1,3]. Rickettsial proliferation within endothelial cells induces direct cell injury [3]. In the acute phase, systemic vasculitis manifests as petechial skin lesions [1]. Ongoing endothelial damage increases permeability of capillaries with resultant microhemorrhage and thrombocytopenic coagulopathy [1]. Continued microvascular leakage may result in acute respiratory distress syndrome and cerebral edema [1,6].

Chronic neurologic sequelae have been observed in patients that suffered a delay in diagnosis and initiation of appropriate antibiotic therapy [7]. Complications include cognitive impairment, meningoencephalitis, deafness, peripheral neuropathy, motor and cerebellar dysfunction, and bladder incontinence [1,8-13]. Despite the systemic nature of SFR vasculitis and the significant morbidity of neuromusculoskeletal complications, no clinical report exists describing the incidence of somatic dysfunction (SD) in SFR. We present a case of previously undiagnosed SFR in a patient referred for Osteopathic Neuromusculoskeletal Medicine (ONMM) evaluation of exacerbated chronic neck and back pain. The incidence of SD is analyzed at seven-week intervals throughout 28 weeks of ONMM care.

## Case presentation

An 81-year-old Caucasian woman living in the southeastern United States was referred for ONMM evaluation by her primary care physician. She presented to the ONMM clinic complaining of exacerbated chronic neck and back pain interfering with sleep for three weeks. She denied fevers, headaches, recent travel, or tick bites. Past medical history included contact dermatitis unresponsive to topical steroids, chronic headaches with negative brain MRI and normal temporal artery biopsy, and chronic disequilibrium unresponsive to balance therapy. The patient was previously evaluated for abdominal bloating and diagnosed with allergy to galactose-alpha-1,3-galactose (alpha-gal), after which she maintained a diet free of mammalian meat. The patient denied tobacco and illicit drug use, consumed one alcoholic beverage every two months, and exercised regularly. She also complained of blurred vision, right hand weakness, a pruritic truncal rash, and urinary incontinence for at least one year.

**Figure 1:**
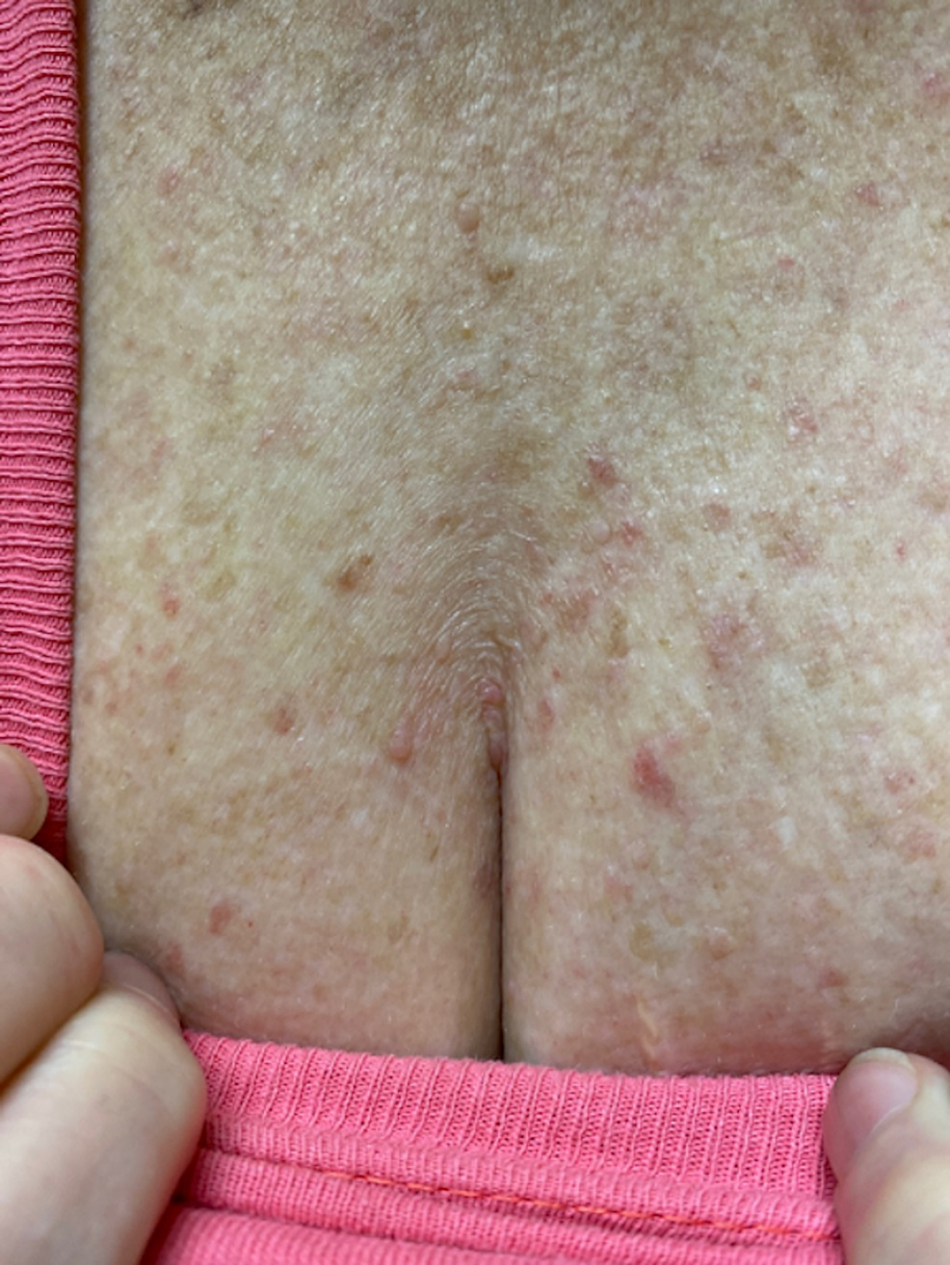
Photograph of the patient's anterior chest. Photograph of the patient’s anterior chest obtained during physical exam was significant for a maculopapular rash which the patient associated with pruritus. Tissue texture was described as nontender, dry, and cool. Similar cutaneous findings were observed along the patient’s back. No rashes were observed on the patient's extremities or scalp.

The patient was in no acute distress and walked into the ONMM clinic without assistance on the initial visit. Vital signs and body mass index were within normal range. Neck exam was significant for diffuse cervical crepitus, rigidity, tenderness, and pain with motion. Musculoskeletal survey revealed decreased passive range of motion throughout the axial skeleton. Skin was warm, dry, and intact with a truncal maculopapular rash (Figure 1).

**Figure 2:**
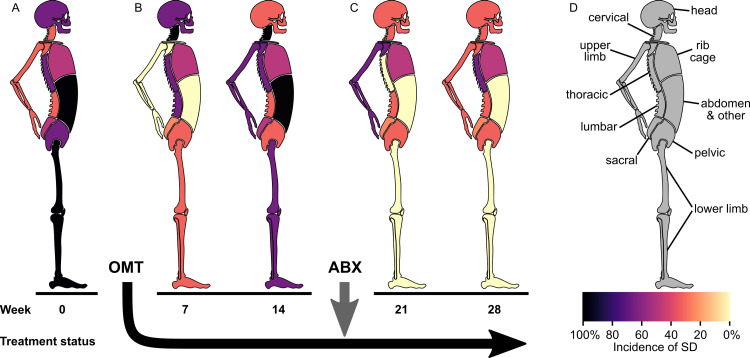
Incidence of somatic dysfunction. A stylized anatomical diagram depicts the incidence of somatic dysfunction (SD) in a patient previously undiagnosed with Spotted Fever Rickettsiosis (SFR). SFR was diagnosed and treated with antibiotic therapy (ABX) between the 14- and 21-week time points. Each shaded body depicts the incidence of SD at seven-week intervals during the 28-week study. A: Incidence of SD diagnosed during week 0, before the start of the osteopathic manipulative treatment (OMT) prescription. B: The incidence of SD diagnosed at week seven (left) and week 14 (right) during the 14-week OMT-only period. C: Incidence of SD diagnosed at week 21 (left) and week 28 (right) during the 14-week post-antibiotic OMT period. D: Anatomical key illustrates the 10 body regions comprising the osteopathic structural exam (top). Incidence of SD is visualized using a colorimetric scale (bottom). Darker colors represent higher incidence, whereas lighter colors represent lower incidence. Specific SD diagnoses recorded per body region were categorized into anatomically defined segments. This approach satisfies the primary osteopathic definition for the term “segment” [14]. Segments included craniosacral, myofascial, transitional skeleton, axial skeleton, and appendicular skeleton. For example, a specific SD diagnosis of “T7ERSr” was categorized under the axial skeleton segment of the thoracic body region, while a diagnosis of “T5-T9 right paraspinal muscle hypertonicity” was categorized under the myofascial segment of the thoracic body region. Incidence of SD was determined by calculating the percentage of total segments per body region with at least one specific SD diagnosis. Results shown each week correspond to a single office visit. The figure is an original anatomical diagram drawn by the lead author of the enclosed report using a free, open-source vector-based graphics software (Inkscape.org).

Initial neurological exam was unremarkable. Baseline osteopathic structural exam (OSE) identified SD in all 10 body regions (Figure 2A). The types of SD found at baseline included myofascial SD in the thorax, pelvic, lower extremity, upper extremity, and abdomen, craniosacral SD was found in the head, sacral, and lower extremity, transitional skeleton SD was found in the head, cervical and lumbar, axial skeleton SD was found in the thorax, sacrum, and ribs, and appendicular skeleton SD was found in the pelvis and lower extremity. Initial management included osteopathic manipulative treatment (OMT) every two weeks on average. OMT was well tolerated, and the patient denied any adverse reaction. 

The OMT-only period spanned 14 weeks after the initial visit. Compared to week 0, incidence of SD during week seven was decreased in the sacral, pelvic, lower limb, upper limb, and abdomen, increased in the cervical and lumbar, and unchanged in the head, thoracic, and rib cage body regions (Figure 2B, left). Compared to week seven, incidence of SD during week 14 was decreased in the head and lumbar, increased in the sacral, lower limb, upper limb, and abdomen, and unchanged in the cervical, thoracic, pelvic, and rib cage body regions (Figure 2B, right). The incidence of SD observed during the 14-week OMT-only period was not significantly different than that observed at initial visit (P = 0.9938, Kruskal-Wallis test/Steel-Dwass method [15]). A one-way, non-parametric method was applied for statistical analysis under the assumption of non-normal data (P = 0.0282, Shapiro-Wilk Goodness-of-Fit) to compare the incidence of SD at three separate time points (initial visit, OMT-only period, post-antibiotic OMT period).

Additional history gathering and physical exam revealed a history of bilateral nocturnal leg cramping, paresthesia, and foot pain. Diabetes screening was negative. Orthopedic evaluation yielded a clinical diagnosis of idiopathic peripheral neuropathy unresponsive to gabapentin. Repeat neurological exam in the ONMM clinic revealed normal mini-mental state, strength, and sensation. Reflexes were abnormal: 1+/4 at right biceps, brachioradialis, and triceps, absent throughout left upper extremity, and absent throughout bilateral lower extremities. Bilateral dysdiadochokinesia was elicited upon finger tapping and heel-to-shin maneuvers.

Electromyography revealed bilateral moderate axonal sensorimotor polyneuropathy. Combined with the lack of significant improvement in SD by week 14, these findings prompted an extensive laboratory workup that was negative except for indirect fluorescent antibody (IFA) RMSF IgG titers that were 1:128, 1:64, and 1:64 during weeks 14, 18, and 24, respectively. Testing for other tick-borne diseases and zoonoses, including Lyme, ehrlichiosis, anaplasmosis, Q fever, and babesiosis, was negative. Testing for blood-borne diseases and sexually transmitted infections, including hepatitis C, human immunodeficiency virus, and syphilis, was negative. Testing for vitamins B9, B12, and D was within references ranges. Testing for thyroid-stimulating hormone, erythrocyte sedimentation rate, and anti-nuclear antibody was also within reference ranges. A chest X-ray revealed chronic, nonspecific parenchymal changes prompting referral to pulmonology where additional testing was conducted and found to be negative, including C-reactive protein, brain-natriuretic peptide, angiotensin-converting enzyme, IgE, rheumatoid factor, anti-cyclic citrullinated peptide, anti-neutrophil cytoplasmic antibodies, typical and atypical anti-neutrophil perinuclear antibodies, 26-item allergen panel, and sputum testing for *Aspergillus fumigatus*, *Micropolyspora faeni*, *Thermoactinomyces vulgaris*, *Thermoactinomyces sacchari*, *Aureobasidium pullulans*, and pigeon serum antibodies. The patient was immediately started on antibiotic therapy for RMSF consisting of oral doxycycline 100 mg twice daily for two weeks. MRI brain revealed nonspecific white matter changes.

The post-antibiotic OMT period spanned weeks 15 to 28. OMT was administered every two weeks on average, was well tolerated, and the patient denied any adverse response. Compared to week 14, incidence of SD during week 21 was decreased in the cervical, thoracic, sacral, lower limb, and abdomen and unchanged in the head, lumbar, pelvic, upper limb, and rib cage body regions (Figure 2C, left). Compared to week 21, incidence of SD during week 28 was decreased in the upper limb, increased in the thorax, and unchanged in the remaining eight body regions (Figure 2C, right). The incidence of SD observed during the 14-week post-antibiotic OMT period was significantly decreased compared to that observed at the initial visit and the OMT-only period (P = 0.0375 and P < 0.0001, respectively). At 21 weeks, the patient’s maculopapular rash had resolved but later returned during week 27. The patient’s cerebellar deficits resolved by week 28, but her abnormal reflexes remained.

## Discussion

This patient presented to the ONMM clinic with the chief complaint of exacerbated chronic neck and back pain. Laboratory workup eventually revealed previously undiagnosed SFR. Significant findings included deficient reflexes, cerebellar signs, and moderate axonal sensorimotor polyneuropathy. Combined with a history of blurry vision, weakness, and urinary incontinence, the patient likely suffered from several neurological sequelae related to SFR that have occurred in similar cases of delayed antibiotic therapy [7,10,16,17]. Only during the post-antibiotic OMT period did we observe a trend towards improvement in the incidence of SD. This observation supports the notion that anatomically diffuse, OMT-refractory SD represents a neuromusculoskeletal complication of SFR that, to date, has not been described in the medical literature. Anatomically diffuse, OMT-refractory SD may, therefore, possibly serve as a marker of underlying systemic disease, such as in the current setting of vasculitis due to SFR or as previously reported in patients with diabetes mellitus [18].

It is unknown exactly when the patient contracted SFR because a convalescent titer was never obtained. Confirmation of tick-borne rickettsial disease by serology requires a fourfold or greater increase in IFA IgG antibody titer between the acute and convalescent phases of illness measured from paired samples 2 to 4 weeks apart [1]. In contrast, diagnosis is supported by one or more samples with a reciprocal IgG titer of ≥ 64 [1]. Therefore, the patient was diagnosed during the non-acute phase of SFR because a fourfold titer increase was not observed.

The extensive workup endured by this patient involved electromyography, numerous blood draws for laboratory analysis, brain MRI imaging, and external specialty referrals. Taken together, these findings support a diagnosis of SFR. A definitive diagnosis of the specific Rickettsia species responsible for the patient’s positive RMSF titers will likely remain unknown for two reasons. One, species-level diagnosis is precluded in the non-acute phase of illness because, as recommended by the Centers for Disease Control and Prevention (CDC), nucleic acid testing for rickettsial DNA will likely be negative outside the acute phase when low numbers of rickettsiae circulate in the blood [1]. Two, species identification is not routinely achieved in clinical practice due to the cross-reactivity of commercial testing platforms that detect serum IgG against rickettsial antigens [1,19]. The CDC acknowledges that mandated rickettsial surveillance programs might not differentiate RMSF from other causes of SFR and recommends reporting new cases of RMSF diagnosed by serology under the general category of SFR [1,2,20].

## Conclusions

This case report provides the first description of SD in SFR. A 14-week trial of OMT for exacerbated neck and back pain failed to significantly reduce the incidence of SD, which prompted additional workup and diagnosis of SFR. Subsequently, the incidence of SD decreased significantly during the post-antibiotic OMT period. This case highlights the utility of periodic graphical assessment of SD to monitor OMT effectiveness.

## References

[1] Biggs HM, Behravesh CB, Bradley KK (2016). Diagnosis and management of tickborne rickettsial diseases: Rocky Mountain spotted fever and other spotted fever group rickettsioses, ehrlichioses, and anaplasmosis - United States a practical guide for health care and public health professionals. MMWR Recomm Rep.

[2] (2022). Centers for Disease Control and Prevention: Epidemiology and statistics. https://www.cdc.gov/rmsf/stats/index.html.

[3] Thorner AR, Walker DH, Petri WA Jr (1998). Rocky Mountain spotted fever. Clin Infect Dis.

[4] Walker DH (1995). Rocky Mountain spotted fever: a seasonal alert. Clin Infect Dis.

[5] Usatine RP, Sandy N (2010). Dermatologic emergencies. Am Fam Physician.

[6] Kaplowitz LG, Robertson GL (1983). Hyponatremia in Rocky Mountain spotted fever: role of antidiuretic hormone. Ann Intern Med.

[7] Sun LR, Huisman TA, Yeshokumar AK, Johnston MV (2015). Ongoing cerebral vasculitis during treatment of Rocky Mountain spotted fever. Pediatr Neurol.

[8] Wright L (1972). Intellectual sequelae of Rocky Mountain spotted fever. J Abnorm Psychol.

[9] Kirk JL, Fine DP, Sexton DJ, Muchmore HG (19901). Rocky Mountain spotted fever: a clinical review based on 48 confirmed cases, 1943-1986.. Med (United States).

[10] Bergeron JW, Braddom RL, Kaelin DL (1997). Persisting impairment following Rocky Mountain spotted fever: a case report. Arch Phys Med Rehabil.

[11] Archibald LK, Sexton DJ (1995). Long-term sequelae of Rocky Mountain spotted fever. Clin Infect Dis.

[12] Buckingham SC, Marshall GS, Schutze GE, Woods CR, Jackson MA, Patterson LE, Jacobs RF (2007). Clinical and laboratory features, hospital course, and outcome of Rocky Mountain spotted fever in children. J Pediatr.

[13] Gorman RJ, Saxon S, Snead OC (19811). Neurologic sequelae of Rocky Mountain spotted fever. Pediatrics.

[14] (2022). American Association of Colleges of Osteopathic Medicine: Glossary of osteopathic terminology. https://www.aacom.org/docs/default-source/insideome/got2011ed.pdf.

[15] Hollander M, Wolfe DA, Chicken E (2015). Nonparametric statistical methods. Wiley.

[16] Danielson L, Vo Q, Alvi M (2019). Microvessel cerebral vasculitis and peripheral neuropathy as neurologic manifestations in Rocky Mountain spotted fever (P5.9-008). Neurology.

[17] Jay R, Armstrong PA (2020). Clinical characteristics of Rocky Mountain spotted fever in the United States: A literature review. J Vector Borne Dis.

[18] Asahi MG, Briganti D, Cam E, Seffinger MA (2020). The role of musculoskeletal disorders in chronic disease: a narrative review. J Am Osteopath Assoc.

[19] Raoult D, Paddock CD (2005). Rickettsia parkeri infection and other spotted fevers in the United States. N Engl J Med.

[20] (2020). Centers for Disease Control and Prevention: For public health officials. https://www.cdc.gov/rmsf/info/index.html.

